# Intermittent Hypoxia Regulates Stem-like Characteristics and Differentiation of Neuroblastoma Cells

**DOI:** 10.1371/journal.pone.0030905

**Published:** 2012-02-17

**Authors:** Vasantha Kumar Bhaskara, Indra Mohanam, Jasti S. Rao, Sanjeeva Mohanam

**Affiliations:** Department of Cancer Biology and Pharmacology, University of Illinois College of Medicine, Peoria, Illinois, United States of America; City of Hope National Medical Center and Beckman Research Institute, United States of America

## Abstract

**Background:**

Neuroblastomas are the most common extracranial solid tumors in children. Neuroblastomas are derived from immature cells of the sympathetic nervous system and are characterized by clinical and biological heterogeneity. Hypoxia has been linked to tumor progression and increased malignancy. Intermittent hypoxia or repeated episodes of hypoxia followed by re-oxygenation is a common phenomenon in solid tumors including neuroblastoma and it has a significant influence on the outcome of therapies. The present study focuses on how intermittent hypoxia modulates the stem-like properties and differentiation in neuroblastoma cells.

**Methods and Findings:**

Cell survival was assessed by clonogenic assay and cell differentiation was determined by morphological characterization. Hypoxia-inducible genes were analyzed by real-time PCR and Western blotting. Immunofluorescence, real-time PCR and Western blotting were utilized to study stem cell markers. Analysis of neural crest / sympathetic nervous system (SNS) markers and neuronal differentiation markers were done by real-time PCR and Western blotting, respectively. Intermittent hypoxia stimulated the levels of HIF-1α and HIF-2 α proteins and enhanced stem-like properties of neuroblastoma cells. In intermittent hypoxia-conditioned cells, downregulation of SNS marker genes and upregulation of genes expressed in the neural crest were observed. Intermittent hypoxia suppressed the retinoic acid-induced differentiation of neuroblastoma cells.

**Conclusions:**

Our results suggest that intermittent hypoxia enhances stem-like characteristics and suppresses differentiation propensities in neuroblastoma cells.

## Introduction

Neuroblastoma is the most common extracranial pediatric solid tumor that is derived from the developing SNS and results from the improper differentiation of neural crest cells [1], [2]. Neuroblastomas show a significant clinical heterogeneity ranging from benign ganglioneuromas to highly aggressive immature tumors with the likelihood of tumor progression varying widely according to age and disease burden at diagnosis [3]. For high-risk neuroblastoma patients, the prospect of long-term survival is dismal despite intensive multimodal therapy [3]. Most solid tumors exhibit regions of hypoxia as a result of inefficient vascular supply by tumor vasculature and high oxygen consumption of rapidly proliferating malignant cells [4]–[6]. Intermittent hypoxia [7]–[10], which is characterized by cyclic periods of hypoxia and reoxygenation, occurs in tumor cells that are dependent on tumor blood vessels having intermittent perfusion fluctuations in blood flow. The occurrence of intermittent hypoxic episodes varies significantly in rapidly growing malignant tumors.

Hypoxia alters neuronal characteristics of human neuroblastoma cells and promotes tumor phenotype with aggressive behavior [11], [12]. Intermittent hypoxia may be associated with generation of a more invasive phenotype of tumor cells and tumor cell dissemination [13]–[15]. Tumor cells that are intermittently hypoxic may affect the response to therapy [9], [16]–[18]. Neuroblastoma is one of the few malignancies that demonstrate spontaneous differentiation and regression to a benign state. Since neuroblastoma with unfavorable prognosis are poorly differentiated, inducing tumor cells to differentiate is an important therapeutic goal in neuroblastoma. In this study, we exposed human neuroblastoma cells to intermittent hypoxia and examined the impact of intermittent hypoxia on SNS progenitor markers, stem cell-like phenotypes and differentiation. In our study, we found that exposing neuroblastoma cells to intermittent hypoxia results in a selection that is highly resistant to differentiation, and exhibits immature neural crest-like and stem-like properties.

## Materials and Methods

### Materials

Anti-mouse Alexa 488, Anti-rabbit Alexa, 594, anti-mouse CD133 were from Invitrogen (Carlsbad, CA). Anti-mouse HIF-1α was from BD Biosciences (San Diego, CA). Anti-mouse HIF-2α was from Novus Biochemicals (Littleton, CO). Antimouse c-kit was from Santa Cruz (Santa Cruz, CA). Anti-mouse tyrosine hydroxylase and β-actin were from Cell Signaling (Danvers, MA). HIF-1α on-target plus smart pool siRNA oligos and on-target plus non-targeting smart pool siRNA oligos were from Dharmacon RNAi Technologies (Lafayette, CO). Anti-mouse Neu-N was from Millipore (Bedford, MA). All-trans-retinoic acid was from Sigma-Aldrich (St.Louis, MO). CD133/1-PE antibodies were from Miltenyi Biotech (Auburn, CA). Rabbit anti-NF-M was from Covance (Princeton, NJ).

### Cell lines

NB1691, a MYCN and MDM2-amplified, chemoresistant [19] human neuroblastoma cell line (provided by Peter Houghton, St. Jude Children's Research Hospital, Memphis, TN) was cultured in DMEM supplemented with 10% heat-inactivated fetal bovine serum and 100 units/mL penicillin, 100 µg/mL streptomycin at 37°C in 5% CO_2_ [20].

### Exposures of cell cultures to intermittent hypoxia treatment

NB1691 cells were exposed to 1, 5 or 10 cycles of hypoxia and normoxia. Each cycle consisted of a period of 24 h in 1% hypoxia followed by 24 h recovery under normoxic conditions. During the reoxygenation period, the medium was changed.

### Exposures of cell cultures to all-trans retinoic acid treatment

Cells were treated with different concentration of all-trans retinoic acid for 24 h and the procedure was performed under dark conditions. No toxicity was observed in the cells due to treatment.

### Transfection of siRNAs

Cells were transfected with 40 nM siRNAs (control and HIF-1α) using Fugene in six-multiwell plates. The cells harvested at 36 h were lysed in cell lysis buffer (Cell Signaling, (Danvers, MA) to obtain proteins for Western blotting analysis. Knockdown of HIF-1α expression was confirmed by Western blot analysis.

### SDS-PAGE and Western blot analysis

Cells were lysed in ice-cold lysis buffer solution containing protease inhibitors and total proteins were extracted as described previously [21]. Samples were subjected to SDS-PAGE and separated proteins were transferred onto nitrocellulose membrane, followed by blocking of membrane with 5% nonfat milk powder (w/v) in Tris- buffered saline (10 mM Tris, 100 mM NaCl, 0.1% Tween 20, pH 7.4) for 1 h at room temperature or overnight at 4°C. Membranes were probed using specific primary antibodies followed by appropriate secondary antibody and enhanced chemiluminescence visualization. Membranes were stripped and reprobed with β-actin antibody as a protein loading control. Nuclear and cytoplasmic extracts were prepared using the Active Motif Nuclear Extraction kit (Active Motif, Carlsbad, CA) according to the manufacturer's instructions.

### Real-time RT-PCR

Total RNA was extracted from cultured cells using TRIzol reagent (Invitrogen, Carlsbad, CA) and reverse-transcribed using the Transcriptor First Strand cDNA synthesis kit (Roche, USA) following the instructions of the manufacturer. Quantitative real-time-PCR reactions were performed in an iCycler iQ Real-Time PCR Detection System (Bio-Rad, Hercules, CA) using the iQ SYBR green supermix (Bio-Rad, Hercules, CA). Results of the real-time PCR data were expressed as relative mRNA expression quantified with Bio-Rad iCycler system software and normalized to β-actin levels.

The forward and reverse primers used were:

HIF-1α, forward (TTCCAGTTACGTTCCTTCGATCA) and

reverse (TTTGAGGACTTGCGCTTTCA);

VEGF, forward (AGGAGGAGGGCAGAATCATCA) and

reverse (CTCGATTGGATGGCAGTAGCT);

Oct-4, forward (GAGAACCGAGTGAGAGGCAACC) and

reverse (CATAGTCGCTGCTTGATCGCT);

CD-133, forward (GCATTGGCATCTTCTATGGTT) and

reverse (CGCCTTGTCCTTGGTAGTGT);

Notch-1, forward (CCGCAGTTGTGCTCCTGAA) and

reverse (ACCTTGGCGGTCTCGTAGCT);

ID-2, forward (TCAGCCTGCATCACCAGAGA) and

reverse (CTGCAAGGACAGGATGCTGAT);

HES-1, forward (AGCGGGCGCAGATGAC) and

reverse (CGTTCATGCACTCGCTGAA);

Neuropeptide tyrosine (NPY), forward (TCCAGCCCAGAGACACTGATT) and

reverse (AGGGTCTTCAAGCCGAGTTCT);

HASH-1, forward (GAGCAGCACACGCGTTATAGTAA) and

reverse (GTGAAGGGACCCGAGCAA);

dHAND, forward (AGAGGAAGAAGGAGCTGAACGA) and

reverse (CGTCCGGCCTTTGGTTTT)

β-Actin, forward (AGAAGGATTCCTATGTGGGCG) and

reverse (CATGTCGTCCCAGTTGGTGAC).

### Immunofluorescence

Cells were fixed for 30 min at −20°C in pre-chilled methanol and permeabilized by 0.3% Triton X-100 in PBS. Cells were incubated overnight at 4°C with the mouse anti-HIF1-α or mouse anti-CD133 antibodies and subsequently incubated with secondary Alexa Fluor 488-conjugated anti-mouse or Alexa Fluor 594-conjugated anti-mouse antibodies, respectively. DNA was visualized by 4′,6-diamidino-2-phenylindole (DAPI) staining. Fluorochromes were visualized with Olympus microscope and imaged. For dual immunofluorescence, incubation with mouse anti-HIF1α and rabbit anti-NF-M was carried out. After washing, cells were incubated with Alexa Fluor 488-conjugated anti-mouse and Alexa Fluor 594-conjugated anti-rabbit IgG secondary antibodies. Finally, cells were counterstained with DAPI for nucleus. Cells were observed under Olympus fluorescent microscope.

### Clonogenic assay

Cells were trypsinized, plated into 100-mm dishes, and incubated at 37°C in a humidified incubator containing 5% CO_2_. After 15 days, cells were stained with crystal violet and colonies having >50 cells were counted as surviving colonies.

### Flow Cytometric analysis

To determine the surface expression of CD133, cells were detached and incubated with the CD133/1-PE antibodies (Miltenyi Biotech, Auburn, CA) according to the manufacturer's instructions. After washing, flow cytometric analysis was done using FACScan (BD Biosciences, San Diego, CA). IgG-PE antibody was used as a control.

### Neurite measurements

Cell morphology was examined at 24 h after 5 µM retinoic acid treatment of human neuroblastoma cells. Cells were considered differentiated if they had one neurite longer than the control group mean neurite length. The length of the longest neurite was measured in at least 500 cells in 10 randomly chosen fields (100× magnification) for each treatment group. Phase-contrast images were taken under bright field using an Olympus CKX41 inverted microscope.

### Statistical analysis

The statistical significance of differences between data sets was determined by using Student's *t*- test. P values less than 0.05 were regarded as significant and were marked with (*) and P<0.01 marked with (**).

## Results

### Regulation of HIF-1α expression in intermittent hypoxia-conditioned neuroblastoma cells

**Figure 1:**
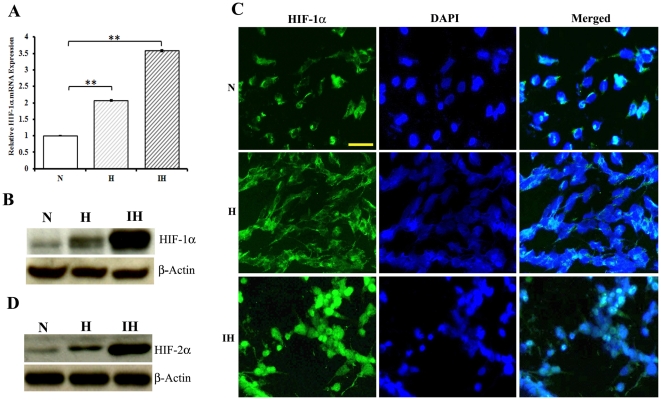
Regulation of HIF-1α expression in intermittent hypoxia-conditioned human neuroblastoma cells. Cells were grown under normoxic conditions (N) or exposed to 1% O_2_ for 24 h (H). Intermittent hypoxia-conditioned cells were derived from NB1691 cells that were exposed to 10 cycles of hypoxia (1% O_2_, 24 h) and reoxygenation. (**A**) Real-time PCR. Total RNA was extracted from neuroblastoma cells using Trizol and cDNA was generated by reverse transcription. Real-time PCRs were done using primers specific to HIF-1α and β-actin. **P<0.01 hypoxia or intermittent hypoxia versus normoxia. (**B**) Cell extracts were assessed for HIF-1α and β-actin by immunoblotting. (**C**) Immunofluorescence. Cells were fixed in ice-cold methanol for 20 min at −20°C. Then, cells were labeled with HIF-1α antibodies and Alexa-488 antimouse-conjugated antibodies. Photomicrographs were taken using Olympus fluorescence microscope. Nuclei were stained with DAPI (bar, 100 µm). (**D**) Cell lysates were probed for HIF-2α and β-actin by western blotting.

We examined the impact of intermittent hypoxia on the abundance of HIF-1α. Intermittent hypoxia-conditioned cells were derived from NB1691 cells that were exposed to 10 cycles of hypoxia and reoxygenation. Each cycle consisted of a period of 24 h in hypoxia (1% O_2_) followed by 24 h recovery under normoxic conditions. Real-time PCRs were done using primers specific to HIF-1α and β-actin. Intermittent hypoxia induced an increase in the abundance of HIF-1α transcript accumulation despite the reoxygenation phases (Fig. 1A and Fig. S1). We also examined the expression of HIF-1α protein by immunofluorescence and immunoblot analyses. Immunofluorescence studies have shown an increase in the levels of HIF-1α protein in hypoxic and intermittent hypoxia-conditioned cells (Fig. 1C). HIF-1α protein is mostly found in the nucleus of intermittent hypoxia-conditioned cells. Immunoblotting experiments revealed that the increase in HIF-1α mRNA transcript observed in response to intermittent hypoxia (Fig. 1A) did translate into an upregulation of protein expression (Fig.1B). An increase in the expression of HIF-2α protein was observed in intermittent hypoxia conditioned human neuroblastoma cells (Fig. 1D). These results indicate that intermittent hypoxia stimulated the expression of HIF-1α and HIF-2α proteins in NB1691 cells.

### Induction of known hypoxia-inducible genes

**Figure 2:**
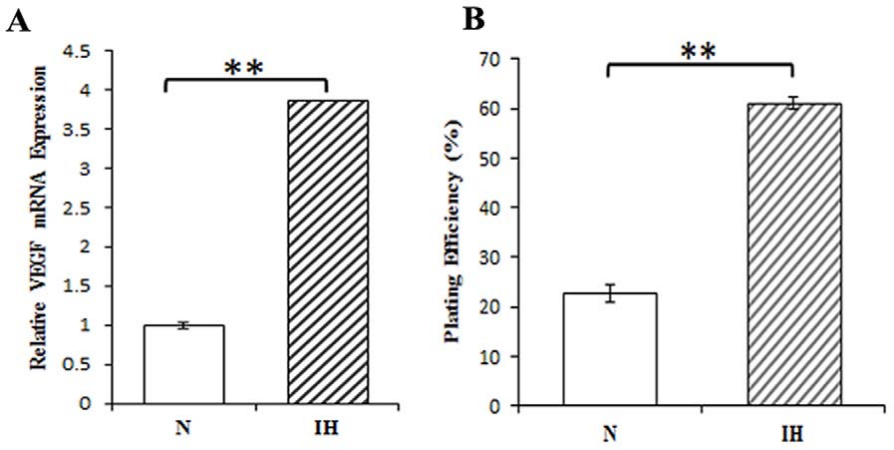
Effects of intermittent hypoxia on VEGF, a hypoxia-response gene and cell survival. (**A**) Real-time PCR. Total RNA was extracted from normoxic (N), and intermittent hypoxia (IH) conditioned neuroblastoma cells using Trizol and cDNA was generated by reverse transcription. Real-time PCRs were done to measure VEGF gene transcript. **P<0.01, intermittent hypoxia versus normoxia. (**B**) Clonogenic assay. Cells were trypsinized, plated into 100-mm dishes, and incubated at 37°C in a humidified incubator containing 5% CO2. After 15 days, cells were stained with crystal violet and colonies having >50 cells were counted as surviving colonies. **P<0.01, intermittent hypoxia versus normoxia.

HIF-1α/β complexes interact with the HIF-responsive element (HRE) of a large number of target genes including VEGF. We therefore used real-time PCR to analyze the expression of VEGF in response to intermittent hypoxia. The expression in intermittent hypoxia versus normoxia control samples shows that VEGF mRNA transcript is up-regulated in NB1619 cells (Fig. 2A).

### Effect of intermittent hypoxia on tumor cell survival

We next used a clonogenic assay to evaluate the effects of intermittent hypoxia. We observed a dramatic gain in survival of intermittent hypoxia conditioned NB1691 cells compared with cells maintained in normoxia (Fig. 2B).

### Effect of intermittent hypoxia on stem-like properties

**Figure 3:**
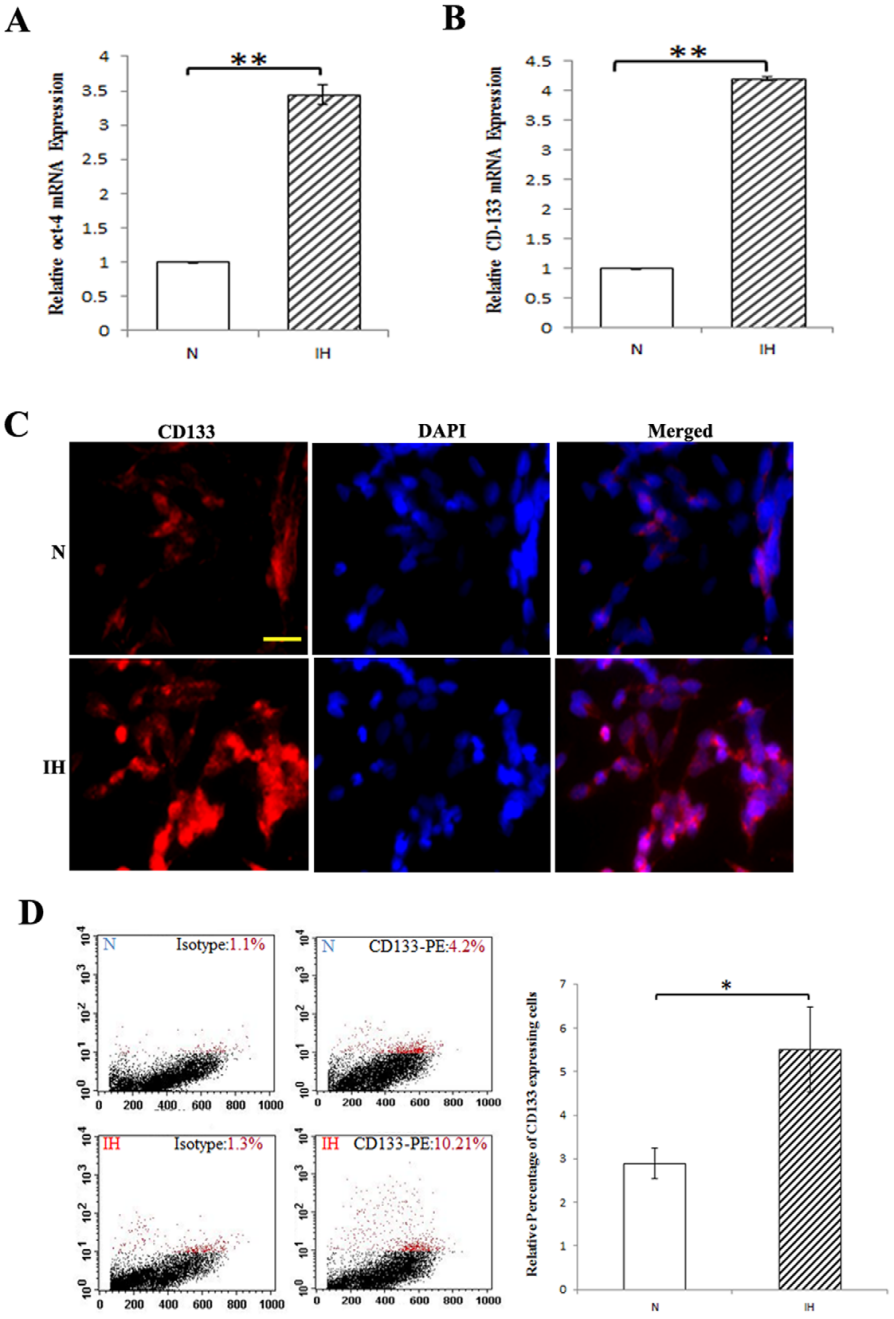
Effects of intermittent hypoxia on stem-like characteristics. Intermittent hypoxia facilitates expression of stem-like characteristics. (**A, B**) Real-time PCR analysis was performed in normoxic (N), and intermittent hypoxia (IH) conditioned neuroblastoma cells using primers specific to Oct-4 and CD133, and normalized to β-actin transcripts. **P<0.01, intermittent hypoxia versus normoxia. (**C**) Immunofluorescence analysis of CD133 expression. Cells were fixed and labeled with CD133 antibodies and Alexa-488 antimouse-conjugated antibodies. Photomicrographs were taken using Olympus fluorescence microscope. Nuclei were stained with DAPI (bar, 100 µm). (**D**) Flow cytometry. Cells were incubated with CD133/1-PE antibodies according to the manufacturer's instructions to determine the surface expression of CD133. After washing, flow cytometry was done using FACScan. IgG-PE antibody was used as a control. A representative flow cytometry analysis is shown. The graph represents the results of experiment done in triplicate.

Hypoxia has been implicated in promoting tumor growth [22]–[24]. Evidence of stem-like cancer cells has been established in various types of cancer, including neuroblastoma [25]–[27]. Since it has been reported that hypoxia exposure alone can increase the stem-like cell population, studies were performed to determine whether intermittent hypoxia would upregulate the expression of stem cell-associated genes in neuroblastoma cells. Using the real-time PCR analysis, we found that the intermittent hypoxia-selected subpopulation displayed an increase in gene transcripts of stem cell markers Oct-4 (Fig. 3A) and CD133 (Fig. 3B) in comparison with the normoxic cells. Further, immunofluorescence and flow cytometry analyses, have confirmed the upregulation of CD133 expression in intermittent hypoxia-conditioned tumor cells (Fig. S2 and Fig. 3C and D). Thus, our data suggest that intermittent exposures of hypoxia and reoxygenation cause enrichment of neuroblastoma cells with stem-like properties.

### Intermittent hypoxia and expression of neural crest /sympathetic nervous system markers

**Figure 4:**
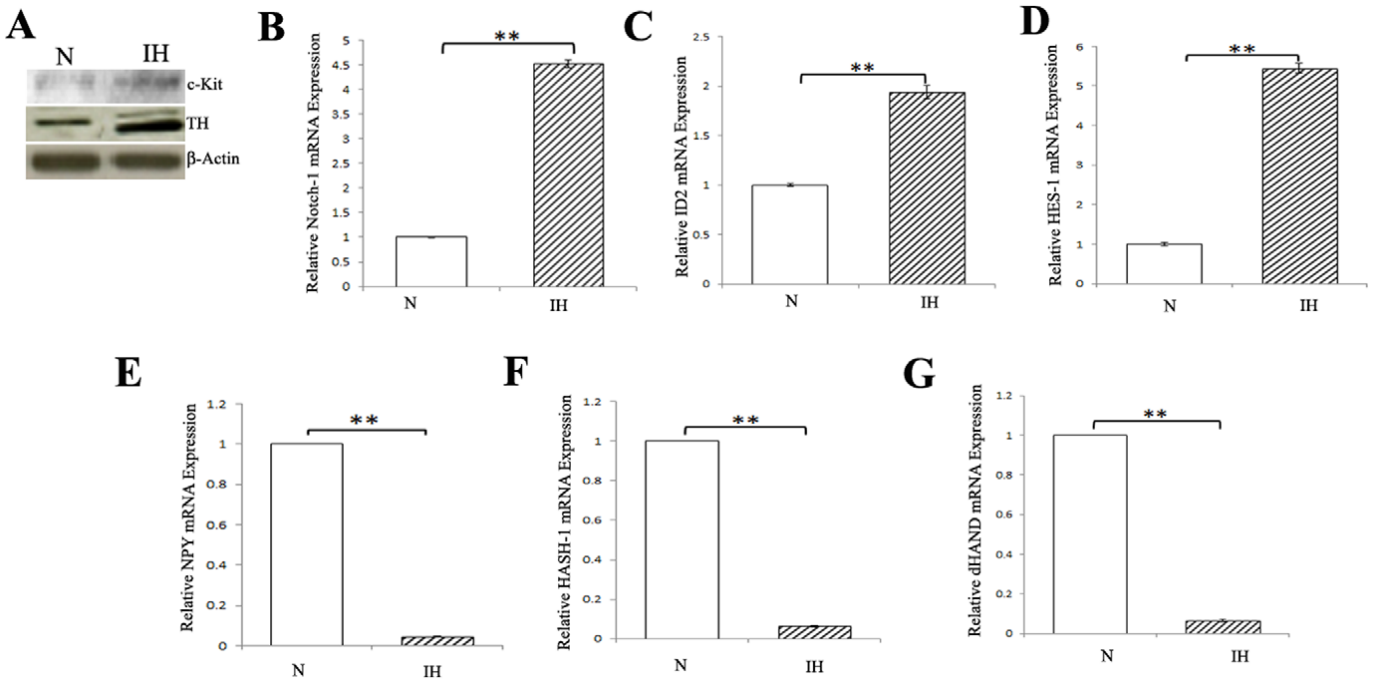
Effects of intermittent hypoxia on neural crest /SNS markers. Upregulation of markers for neural crest genes. (**A**) Western Blotting. Cell lysates of normoxic (N) and intermittent hypoxia (IH) conditioned neuroblastoma cells were analyzed by western blotting for the levels of c-Kit and TH. Real-time PCR. PCR analysis was performed in normoxic (N) and intermittent hypoxia (IH) conditioned neuroblastoma cells using primers specific to Notch-1 (**B**), ID2 (**C**) and HES-1(**D**) gene transcripts. **P<0.01, intermittent hypoxia versus normoxia. Downregulation of SNS markers. Real-time PCR. PCR analysis was performed in normoxic (N) and intermittent hypoxia (IH) conditioned neuroblastoma cells using primers specific to NPY (**E**), HASH-1(**F**) and dHAND (**G**). **P<0.01, intermittent hypoxia versus normoxia.

Hypoxia has been linked to creation of a microenvironment enriched in poorly differentiated tumor cells [28]. HIF-1α and HIF-2α have been linked to an aggressive tumor phenotype by promoting the processes essential for tumor growth as well as blocking differentiation [29], [30]. Studies were performed to determine whether intermittent hypoxia modulates the expression of neural crest genes. Immunoblot analysis has shown elevated expression levels of tyrosine hydroxylase(TH) and c-Kit proteins in intermittent-hypoxia conditioned cells (Fig. 4A). As further evidence, real-time PCR analysis demonstrated an increase in transcripts of neural crest markers Notch-1, ID2 and HES-1in intermittent hypoxia-conditioned cells (Fig. 4B, C, D). We also studied the effects of intermittent hypoxia on the differentiation status of neuroblastoma cells by examining the expression of the sympathetic neuronal peptide neurotransmitter gene, NPY. The expression levels of HASH-1 and dHAND genes that are associated in early sympathetic lineage specification were determined by real-time PCR. Our results show that the expression levels of NPY, HASH-1and dHAND were found decreased in intermittent hypoxia conditioned tumor cells (Fig. 4E,F,G). These observations reveal that intermittent hypoxia-exposed cultured human neuroblastoma cells exhibit downregulation of SNS marker genes, such as the neurofilament genes NPY, HASH-1 and dHAND, as well as upregulation of genes expressed in the neural crest, e.g., ID2, HES-1, c-Kit and Notch-1.

### Effect of retinoic acid on neuroblastoma cells

It has been known that neuroblastoma cells can be induced to differentiate into neuron-like cells by retinoic acid [31], [32]. We sought to examine the effects of retinoic acid on neuronal properties. Here NB1691 cells were treated with various concentrations of retinoic acid and investigated for the expression of neuronal markers including Neu N and NF-M. Protein levels of neuronal markers in untreated and retinoic acid treated NB1691 cells were compared by Western blot analysis (Fig. S3). We observed a significant increase in the expression of NF-M and Neu N in NB1691 cells differentiated upon retinoic acid treatment. Hypoxia reduced the levels NF-M and Neu N proteins induced by retinoic acid in neuroblastoma cells. HIF-1α levels increased under hypoxia; however retinoic acid reduced hypoxia-induced HIF-1α (Fig. S3).

**Figure 5:**
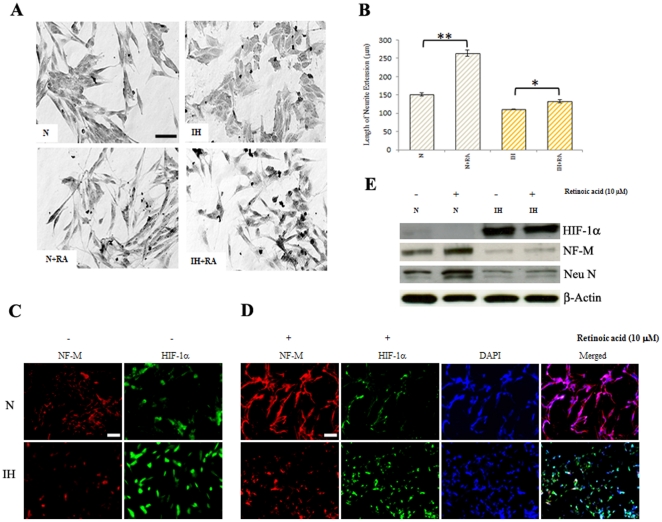
Effect of retinoic acid on human neuroblastoma cells. (**A**) Normoxic (N) and intermittent hypoxia (IH) conditioned neuroblastoma cells were treated with 5 µm retinoic acid for 24 h and cell morphology was examined. Phase-contrast images were taken under bright field using an Olympus CKX41 inverted microscope (bar, 50 µm). (**B**) Graphic illustration of quantification of neurite lengths of normoxic and intermittent-hypoxia conditioned neuroblastoma cells treated with 5 µm retinoic acid. *P<0.05 **P<0.01, retinoic acid-treated versus untreated. (**C**) Immunofluorescence. Cells were fixed and incubated with primary antibodies for NF-M or HIF-1α. Then cells were washed in PBS and incubated with secondary antibodies, Alexa Fluor 488-conjugated anti-mouse IgG (HIF-1α) or Alexa Fluor 594-conjugated anti-rabbit IgG (NF-M) (bar, 100 µm). (**D**) Dual Immunofluorescence. Cells were treated with 10 µM retinoic acid for 24 h fixed and incubated with primary antibodies for NF-M or HIF-1α. Then cells were washed in PBS and incubated with secondary antibodies, Alexa Fluor 488-conjugated anti-mouse IgG or Alexa Fluor 594-conjugated anti-rabbit IgG. Nuclei were stained with DAPI. Photomicrographs were taken using Olympus fluorescence microscope (bar, 100 µm). (**E**) Western blotting: Cells were treated with 10 µM retinoic acid for 24 h. Cell lysates were analyzed for the levels of HIF-1α, NF-M and Neu N proteins by western blotting. β-actin served as loading control.

Next, neuroblastoma cells were treated with retinoic acid and the cell morphology was examined. Fig. 5A shows extension of neurites, a typical neuronal phenotype suggesting that NB1691 cells undergo differentiation upon treatment with retinoic acid. An analysis of morphological differentiation was obtained by measuring neurite length (Fig. 5A). The mean neurite length was significantly reduced in intermittent hypoxia-conditioned cells (Fig. 5B). Further, the potentiation of neuronal differentiation by retinoic acid was significantly less in intermittent hypoxia-conditioned cells compared with the cells maintained in normoxia.

To confirm the changes in the expression of neuronal markers revealed by western-blot analysis, an indirect double-immunofluorescence stain with anti-NF-M and anti- HIF-1α was performed in NB1691 cells with and without retinoic acid treatment. Fluorescent intensity of NF-M and HIF-1α was compared between parental and retinoic acid treated NB1960 cells (Fig. 5C). We observed a significant increase in fluorescent intensity in NF-M and a decrease in the intensity of HIF-1α in cells treated with retinoic acid. Intermittent hypoxia conditioned cells exhibit decreased NF-M intensity and higher HIF-1α intensity compared with control. No significant changes in the intensity of NF-M and HIF-1α were observed in intermittent hypoxic cells untreated and treated with retinoic acid (Fig. 5D).

To validate the changes in the expression of neuronal markers revealed by immunocytochemical studies, western blot analysis was then performed. A decrease in NF-M and Neu N was found in intermittent hypoxia-conditioned cells. Retinoic acid upregulated NF-M and Neu N protein levels in normoxic cells; however, no increase was observed in intermittent hypoxia-conditioned cells (Fig. 5E).

### Effect of HIF-1α knockdown on differentiation of neuroblastoma cells

**Figure 6:**
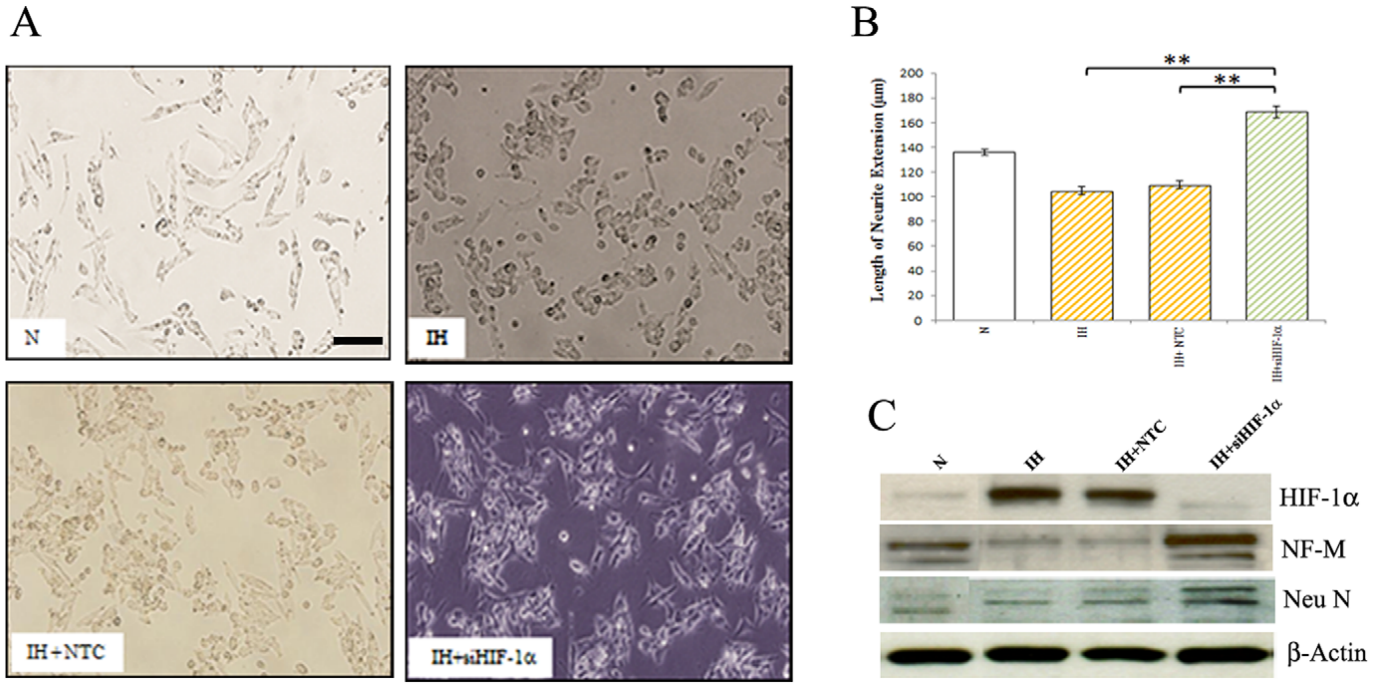
Effect of HIF-1α siRNA on differentiation of human neuroblastoma cells. (**A**) Normoxic (N) and intermittent hypoxia (IH) conditioned neuroblastoma cells were treated with non-targeted control (NTC) or HIF-1α siRNA smart pool for 36 h and phase-contrast images were taken under bright field using an Olympus CKX41 inverted microscope (bar, 50 µm). (**B**) Graph illustrates quantification of neurite lengths of cells treated with NTC or HIF-1α siRNA. **p<0.01, intermittent hypoxia conditioned cells treated with HIF-1α siRNA versus NTC or untreated. (**C**) Western blotting. Cells were treated with NTC or HIF-1α siRNA smart pool. After 36 h, cells were lysed and cell extracts were subjected to western blotting analysis for HIF-1α, NF-M and Neu N.

We addressed the possible role of the IH on differentiation of neuroblastoma cells. To analyze the role of the HIF-1α in the regulation of differentiation of neuroblastoma cells, the HIF-1α was silenced by transfection of specific HIF-1α siRNA. The efficiency of siRNA knock-down was assessed by immunoblotting with antibodies against HIF-1α. As expected, the HIF-1α protein was markedly reduced in the siRNA-transfected cells. An analysis of morphological differentiation has shown an increase in neuronal differentiation in intermittent hypoxia-conditioned cells treated with HIF-1α siRNA under hypoxia (Fig. 6A, B). To further explore the effect of the HIF-1α on differentiation, we investigated the protein levels of Neu N and NF-M. As expected, an increase in NF-M and Neu N protein levels were found in intermittent hypoxia-conditioned cells treated with HIF-1α siRNA (Fig. 6C).

## Discussion

Neuroblastic tumors are characterized by extreme clinical and pathological heterogeneity [33]. Hypoxia is widespread in solid tumors as a consequence of microregional fluctuations in perfusion as well as poorly structured tumor vasculature [4]–[6]. Like other solid tumor cells, neuroblastoma cells are able to adapt to hypoxia by modulating their phenotype. Intermittent hypoxia is described as a more representative picture of the oxygen tension of the environment in tumors rather than a permanent exposure to low oxygen levels. Many previous studies focused on acute or chronic hypoxia, but intermittent hypoxia also plays an important role in solid tumors. Metastasis-associated genes were found significantly upregulated in hypoxic cells sorted from tumors of intermittent hypoxia treated mice compared with hypoxic cells derived from tumors exposed to normoxia [34].

The effects of intermittent hypoxia on neuroblastoma cells remain unclear needing further investigations. Hypoxia, when followed by reoxygenation has been demonstrated to induce oxidative stress in cancer cells and promotes tumor development [35], [36]. Tumor cells have been exposed to a wide range of periods of hypoxia from hours to days in various cell culture studies of intermittent hypoxia [13], [37]–[41]. Our protocol of intermittent hypoxia was also based on other cell culture studies and we selected a sequence of hypoxic and normoxic intervals of 24 h in an effort to replicate the hypoxic-resistant intratumoral environment *in vitro*.

Expression of HIF-1α increased progressively after at 5 and 10 cycles of hypoxia and reoxygenation as evidenced by immunoblotting data (Fig. S1). Studies have shown that the reoxygenation of hypoxic tumor cells can also result in free radical formation, leading to the nuclear accumulation of HIF-1α [42]. The nuclear accumulation of HIF-1α under intermittent hypoxia could explain the increase in HIF-1 transcriptional activity as observed by the increase in target gene mRNA levels such as VEGF which is associated with tumor angiogenesis [43]. Different roles of HIF-1α and HIF-2α in hypoxic gene regulation have been demonstrated in various cell types [44], [45]. In our study, it was found that, like HIF-1α, HIF-2α level was also increased. Our observation of an increased expression of HIF-2 α in response to intermittent hypoxia in neuroblastoma cells is in agreement with other studies showing induction of HIF-2α by hypoxia [11], [46]. However, Holmquist et al [38] have shown that prolonged hypoxia diminishes HIF-1α protein. This inconsistency could be explained either by difference in cell type, or by different mechanisms switched on during intermittent hypoxia in comparison to chronic hypoxia. Clonogenic assay reveals an increased survival of intermittent hypoxia-conditioned neuroblastoma cells. These results support that the stabilization of HIF-1α and HIF-2α enhanced tumor cell survival under intermittent hypoxia.

We next examined the influence of intermittent hypoxia on enhancement of stem-like properties of cells. Our real-time PCR analysis revealed an increase in the expression of stem cell markers CD133 and Oct-4 in intermittent hypoxia conditioned cells compared with cells grown under normoxia. Both Immunofluorescence and flow cytometry analysis also confirmed the upregulation of CD133 in intermittent hypoxia conditioned cells. Further, we observed CD133 expression is localized in membrane and cytosolic compartments of tumor cells. By immunohistochemical staining, Tong et al [47] have reported that CD133 was mainly expressed in the cytoplasm of tumor cells of neuroblastoma patients. Studies have shown that hypoxia can drive a phenotype that can increase the stem-like phenotype or the number of stem-like cancer cells to ensure survival of the tumor [26], [27], [48]. Knockdown of HIF-1α abrogated the hypoxia-mediated CD133-positive cancer stem cell expansion in gliomas [49]. It has been shown that HIF-2α protein is expressed consistently higher in glioma stem cells than in matched non-stem cancer cells or normal neural progenitors indicating that HIF-2α induction is restricted to cancer stem cells [50]. Our results support that intermittent hypoxia could expand selectively stem-like subpopulation in the neuroblastoma cells in part through upregulation of HIF-1α and HIF-2α.

Neuroblastoma cell lines exhibit many of the cell phenotypes characteristic of the developing neural crest cells such as cellular heterogeneity, plasticity and transdifferentiation capacity [51]. In our studies, crest cell markers such as c-kit, Notch-1, HES-1, and ID2 were found to be increased reflecting immature state in cells exposed to intermittent hypoxia. Interestingly, Notch1, ID2, and HES1 were proposed as mediators of dedifferentiation. The Notch signaling pathway has been shown to inhibit neuroblastoma tumor cell differentiation [52] and Notch1 expression has been shown to be associated with high-risk tumor features and poor prognosis in a cohort of children with neuroblastoma [53]. The expression of genes immediately downstream of Notch, such as HES [54] was altered by hypoxia. [55]. It has been shown that ID2 is involved in normal neural crest development. Hypoxia induces ID2 expression and hypoxia-induced ID2 expression play a significant role in dedifferentiation of hypoxic neuroblastoma cell [56]. Intermittent hypoxia also caused a decrease in the expression of SNS neuronal lineage-specific marker genes, including NPY, Hash-1 and dHAND. Others have reported that neuroblastoma cells lost their neuronal/ neuroendocrine features and gained immature, neural crest-like phenotype upon exposure to hypoxia [38], [57].

Induced differentiation of transformed cells into mature phenotypes represents a promising strategy in recent antitumor therapy .The treatment of neuroblastoma patients with retinoids during maintenance therapy has increased survival rates [58], [59]. In the current study, we identified a role for intermittent hypoxia in retinoic acid-mediated induction of neuronal differentiation in neuroblastoma cells. We observed that a decrease in differentiation was observed in retinoic acid-treated cells exposed to intermittent hypoxia accompanied by reduced neurite outgrowth and the suppression of neuronal differentiation markers, NF-M and Neu N. On the other hand, inhibition of HIF-1α expression by transfection of HIF-1 siRNA promoted neuronal differentiation in intermittent hypoxia conditioned cells as measured by up-regulation of NF-M and Neu N, as well as morphological changes. Our results suggest that HIF-1α plays a marked role in suppressing retinoic acid-induced differentiation of neuroblastoma cells exposed to intermittent hypoxia.

Taken together, multiple cycles of hypoxia and reoxygenation provide selective advantage to neuroblastoma cells and can lead to complex changes which are associated with enhanced stem-like features, immature neural-crest-line phenotype and decreased retinoic acid-induced differentiation. Indeed, targeting oxygen response pathways, such as HIF-1α and HIF-2α, are promising strategies for suppressing neuroblastoma development and improving outcomes of therapies.

## Supporting Information

Figure S1 **HIF-1α expression in intermittent hypoxia-conditioned neuroblastoma cells.** Intermittent hypoxia-conditioned cells were derived from NB1691 cells that were exposed to 1, 5 or10 cycles of hypoxia and reoxygenation. Each cycle consisted of a period of 24 h in hypoxia (1% O_2_) followed by 24 h recovery under normoxic conditions. Cell extracts were assessed for HIF-1α and β-actin by western blotting.
(TIF)

Figure S2 **Dual Immunofluorescence.** Normoxic (N) and intermittent hypoxia (IH) conditioned neuroblastoma cells were incubated with primary antibodies for CD133 or HIF-1α. Then cells were washed in PBS and incubated with secondary antibodies, Alexa Fluor 488 or 594-conjugated anti-mouse IgG. Nuclei were stained with DAPI (bar, 100 µm).
(TIF)

Figure S3 **Effect of retinoic acid on human neuroblastoma cells.** Western blotting: NB1691 cells were treated with various concentrations of retinoic acid for 24 h under hypoxia or normoxia. Cell lysates were analyzed for the levels of HIF-1α, NF-M and Neu N proteins by western blotting.
(TIF)
