# Correction: Intermittent hypoxia regulates stem-like characteristics and differentiation of neuroblastoma cells

**DOI:** 10.1371/journal.pone.0328935

**Published:** 2025-07-24

**Authors:** Vasantha Kumar Bhaskara, Indra Mohanam, Jasti S. Rao, Sanjeeva Mohanam

The panels presented in Fig 5E and Fig 6C were prepared using spliced blot data. Specifically, lanes were removed between lanes 2 and 3 in all four panels in Fig 5E, and lanes were removed between lanes 1 and 2 in all four panels in Fig 6C. The Figs 5 and 6 have been updated to clearly mark the splice lines.

**Fig 5:**
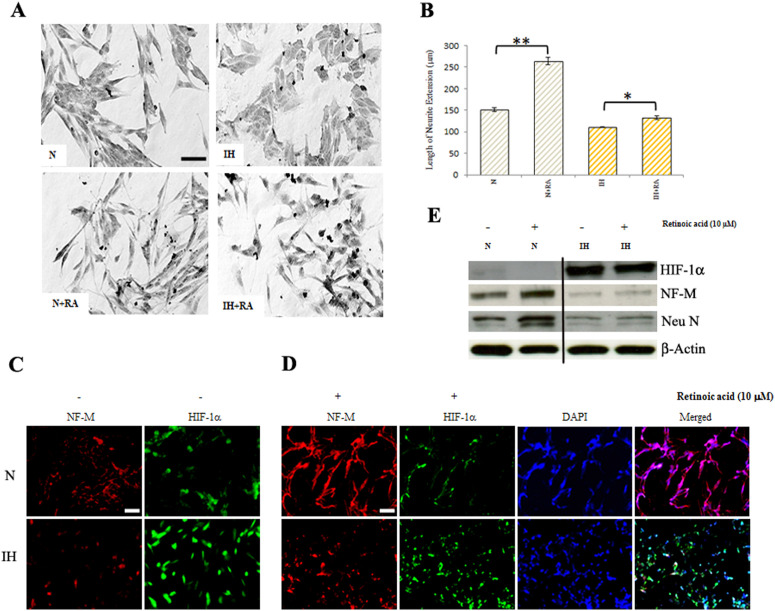
Effect of retinoic acid on human neuroblastoma cells. (A) Normoxic (N) and intermittent hypoxia (IH) conditioned neuroblastoma cells were treated with 5 µm retinoic acid for 24 h and cell morphology was examined. Phase-contrast images were taken under bright field using an Olympus CKX41 inverted microscope (bar, 50 µm). (B) Graphic illustration of quantification of neurite lengths of normoxic and intermittent-hypoxia conditioned neuroblastoma cells treated with 5 µm retinoic acid. *P < 0.05 **P < 0.01, retinoic acid-treated versus untreated. (C) Immunofluorescence. Cells were fixed and incubated with primary antibodies for NF-M or HIF-1α. Then cells were washed in PBS and incubated with secondary antibodies, Alexa Fluor 488-conjugated anti-mouse IgG (HIF-1α) or Alexa Fluor 594-conjugated anti-rabbit IgG (NF-M) (bar, 100 µm). (D) Dual Immunofluorescence. Cells were treated with 10 µM retinoic acid for 24 h fixed and incubated with primary antibodies for NF-M or HIF-1α. Then cells were washed in PBS and incubated with secondary antibodies, Alexa Fluor 488-conjugated anti-mouse IgG or Alexa Fluor 594-conjugated anti-rabbit IgG. Nuclei were stained with DAPI. Photomicrographs were taken using Olympus fluorescence microscope (bar, 100 µm). (E) Western blotting: Cells were treated with 10 µM retinoic acid for 24 h. Cell lysates were analyzed for the levels of HIF-1α, NF-M and Neu N proteins by western blotting. β-actin served as loading control.

**Fig 6:**
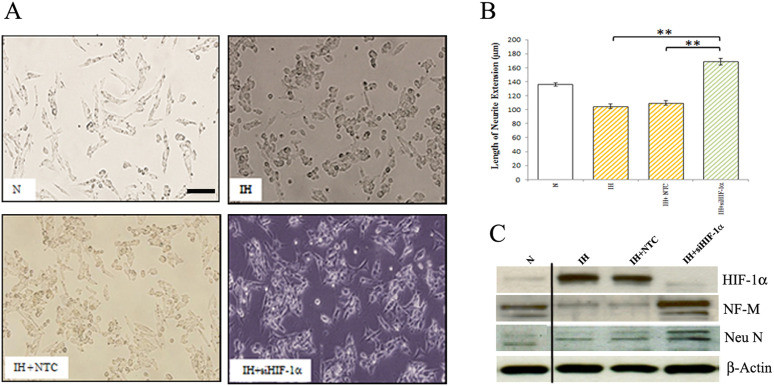
Effect of HIF-1 α siRNA on differentiation of human neuroblastoma cells. (A) Normoxic (N) and intermittent hypoxia (IH) conditioned neuroblastoma cells were treated with non-targeted control (NTC) or HIF-1α siRNA smart pool for 36 h and phase-contrast images were taken under bright field using an Olympus CKX41 inverted microscope (bar, 50 µm). (B) Graph illustrates quantification of neurite lengths of cells treated with NTC or HIF-1α siRNA. **p < 0.01, intermittent hypoxia conditioned cells treated with HIF-1α siRNA versus NTC or untreated. (C) Western blotting. Cells were treated with NTC or HIF-1α siRNA smart pool. After 36 h, cells were lysed and cell extracts were subjected to western blotting analysis for HIF-1α, NF-M and Neu N.

The available underlying blot data for Figs 5E and 6C are provided in S1 and S2 File. The quantitative data underlying the graphs presented in Figs 1-5 are provided in S3 File. The available quantitative data underlying Fig 6B are provided in S4 File.

## Supporting information

S1 File Cropped blot data underlying Fig 5E. (TIF)

S2 File Cropped blot data underlying Fig 6C. (TIF)

S3 File Quantitative data underlying graphs published in Figs 1–5. (XLSX)

S4 File Available quantitative data underlying Fig 6B. (XLSX)
