# Supplementary figures and images for: Intermittent Hypoxia Regulates Stem-like Characteristics and Differentiation of Neuroblastoma Cells
Source: PLoS One. 2012 Feb 17;7(2):e30905. doi: 10.1371/journal.pone.0030905 (PMC3281893; doi:10.1371/journal.pone.0030905)

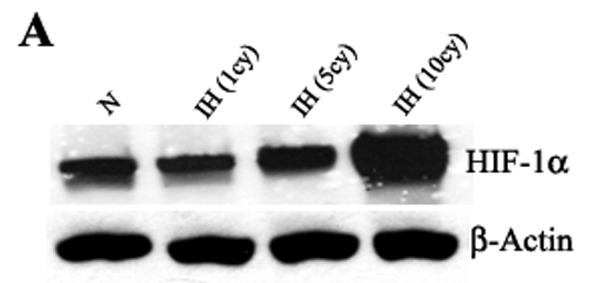

Supplement: Figure S1 — HIF-1α expression in intermittent hypoxia-conditioned neuroblastoma cells. Intermittent hypoxia-conditioned cells were derived from NB1691 cells that were exposed to 1, 5 or10 cycles of hypoxia and reoxygenation. Each cycle consisted of a period of 24 h in hypoxia (1% O2) followed by 24 h recovery under normoxic conditions. Cell extracts were assessed for HIF-1α and β-actin by western blotting. (TIF) [file pone.0030905.s001.tif]

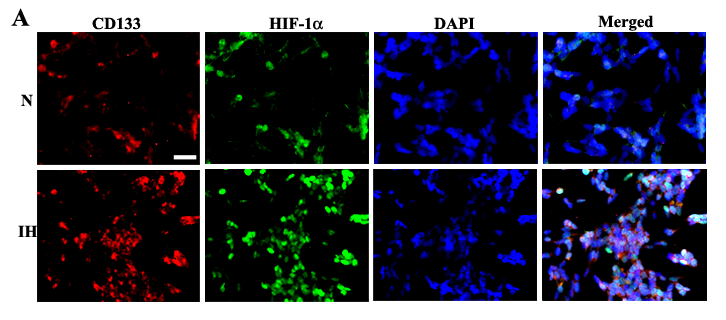

Supplement: Figure S2 — Dual Immunofluorescence. Normoxic (N) and intermittent hypoxia (IH) conditioned neuroblastoma cells were incubated with primary antibodies for CD133 or HIF-1α. Then cells were washed in PBS and incubated with secondary antibodies, Alexa Fluor 488 or 594-conjugated anti-mouse IgG. Nuclei were stained with DAPI (bar, 100 µm). (TIF) [file pone.0030905.s002.tif]

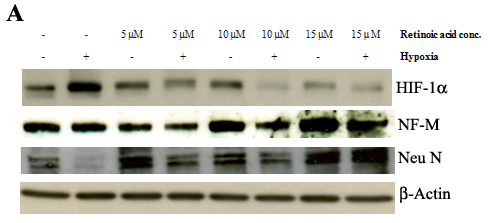

Supplement: Figure S3 — Effect of retinoic acid on human neuroblastoma cells. Western blotting: NB1691 cells were treated with various concentrations of retinoic acid for 24 h under hypoxia or normoxia. Cell lysates were analyzed for the levels of HIF-1α, NF-M and Neu N proteins by western blotting. (TIF) [file pone.0030905.s003.tif]
