# Supplementary figures and images for: Correction: Intermittent hypoxia regulates stem-like characteristics and differentiation of neuroblastoma cells
Source: PLoS One. 2025 Jul 24;20(7):e0328935. doi: 10.1371/journal.pone.0328935 (PMC12289004; doi:10.1371/journal.pone.0328935)

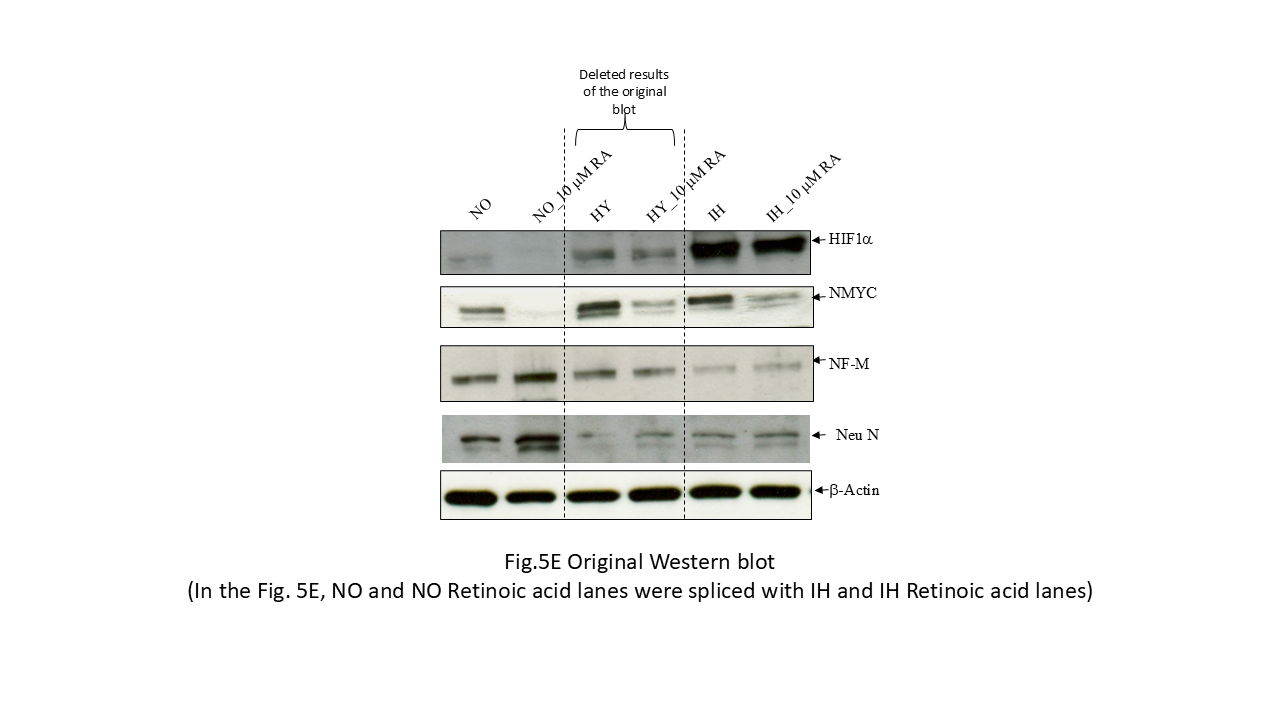

Supplement: S1 File — (TIF) [file pone.0328935.s001.tif]

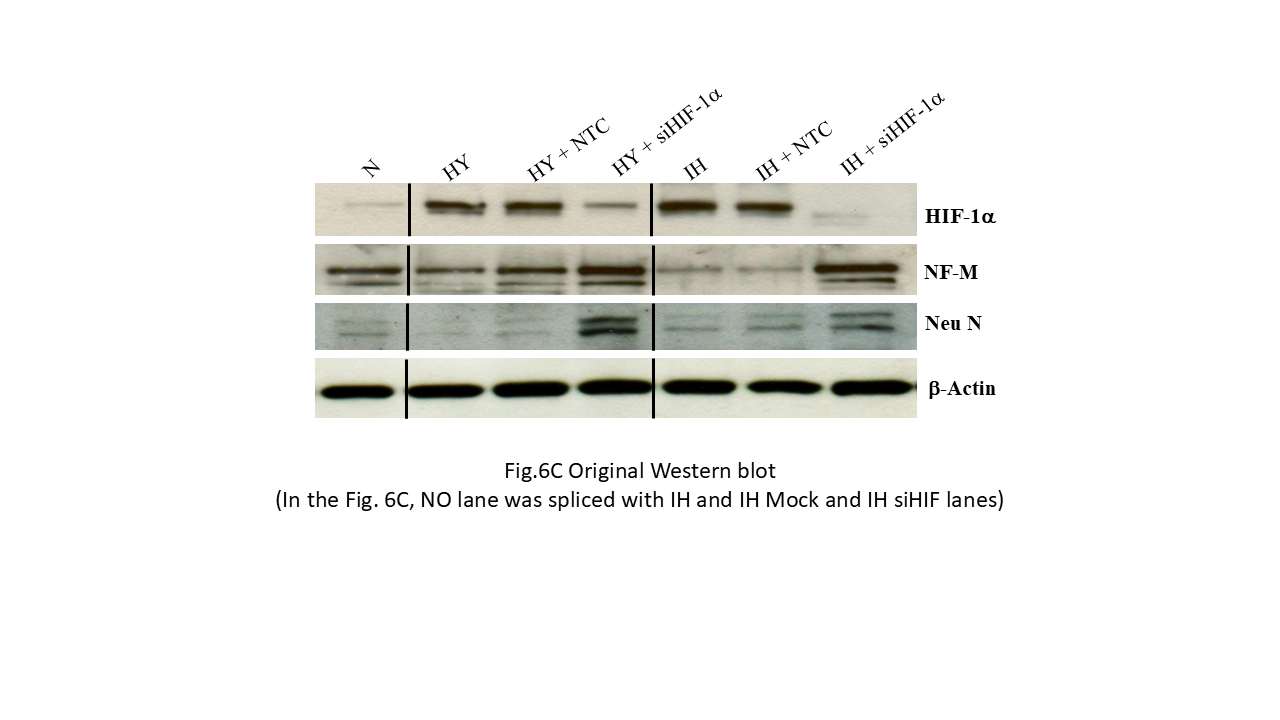

Supplement: S2 File — (TIF) [file pone.0328935.s002.tif]
